# Causal associations of birth body size and adult body size with systemic lupus erythematosus: a bidirectional mendelian randomization study

**DOI:** 10.3389/fgene.2024.1368497

**Published:** 2024-05-06

**Authors:** Juan Peng, Huizi Wang, Yanjuan Li, Xudong Dong

**Affiliations:** ^1^ Medical Faculty, Kunming University of Science and Technology, Kunming, Yunnan, China; ^2^ Obstetrics Department, The First People’s Hospital of Yunnan Province, Affiliated Hospital of Kunming University of Science and Technology, Kunming, Yunnan, China; ^3^ Faculty of Environmental Science and Engineering, Kunming University of Science and Technology, Kunming, Yunnan, China

**Keywords:** mendelian randomization, birth weight, height, causality, systemic lupus erythematosus

## Abstract

**Objective:**

Body size is associated with the onset of systemic lupus erythematosus (SLE). However, the evidence for this association is inconclusive. In this study, we aimed to investigate the causal relationship between body size and SLE.

**Method:**

We performed a bidirectional Mendelian randomization (MR) analysis that utilized summary statistics sourced from genome-wide association study (GWAS) data obtained from the IEU Open GWAS project website. The inverse variance weighting (IVW) method was used to evaluate the causality, and four additional MR methods were used to supplement the IVW results. Sensitivity analyses were performed using the Cochran’s Q test, MR-Egger regression, leave-one-out analysis, and the Mendelian Randomization Pleiotropy RESidual Sum and Outlier (MR-PRESSO) global test.

**Results:**

In the forward direction analysis, the IVW model demonstrated that birth weight (odds ratio (OR), 1.811; 95% confidence interval (CI), 1.174–2.793; *p* < 0.05) and adult height (OR, 1.225; 95% CI, 1.046–1.434; *p* < 0.05) were positively associated with SLE. Four additional MR scans were performed parallel to the IVW results. Conversely, SLE was a weak causal factor for increased height (OR, 1.010; 95% CI, 1.002–1.018; *p* < 0.05) using the IVW method. Heterogeneity, MR-Egger intercept, and leave-one-out analyses indicated that the results were robust. The MR-PRESSO suggested the presence of pleiotropy. Following the exclusion of instrumental variables (IVs) inducing pleiotropy, subsequent MR analysis yielded consistent results, thereby reinforcing the robustness of our findings.

**Conclusion:**

Positive causal associations were observed between birth weight, adult height, and SLE incidence. In the reverse analysis, SLE was a weak causal factor for adult height.

## 1 Introduction

Systemic lupus erythematosus (SLE) is a chronic autoimmune disease that often occurs in young women, is characterized by the involvement of different organ systems and immune abnormalities, and has high morbidity and mortality rates (Barber et al., 2021). SLE is believed to arise from the disruption of immune tolerance in genetically predisposed individuals under the sustained influence of various exogenous pathogenic factors, including cigarette smoking, viral infections, estrogenic exposure, and ultraviolet radiation (Leffers et al., 2019). SLE-associated autoantibodies can be detected many years before the diagnosis, suggesting a cumulative effect of these factors (Arbuckle et al., 2003; Olsen and Karp, 2014). Adipose tissue accumulation may be a contributing factor, as recent evidence suggests that adult obesity is associated with an increased risk of SLE (Tedeschi et al., 2017). An increase in childhood obesity rates in Western countries has been observed alongside an increase in SLE prevalence (Ng et al., 2014; Rees et al., 2017). Previous studies demonstrated a significant correlation between birth weight and adult height (Richardson et al., 2019). According to the Developmental Origins of Health and Disease (DOHaD) theory, environmental exposure during early life (especially in the in-utero period) can permanently influence health and vulnerability to disease in later life (Hoffman et al., 2021). Therefore, early-life and adult body size may be important contributors to the development of SLE.

However, few studies have investigated the relationship between body size and SLE. Furthermore, existing reports on the correlation between birth weight, an indicator of intrauterine growth, and SLE prevalence have yielded inconsistent results. A comprehensive cohort study showed a positive association between birth weight of ≥10 pounds and SLE occurrence in women (Simard et al., 2008). By contrast, another cohort study reported an association between SLE and low birth weight (Parks et al., 2016). Several case–control studies have found that birth weight is not associated with SLE development (Coleman et al., 2005; Arkema and Simard, 2015). Further research is needed to investigate the association between body size and SLE risk.

In traditional epidemiological studies, the connection between exposure and outcome might be affected by unmeasured confounding factors and reverse causation, potentially hindering the determination of causality. In recent years, Mendelian randomization (MR) has emerged as a valuable approach for causality studies using genome-wide association study (GWAS) data (Skrivankova et al., 2021). MR leverages randomly allocated allelic variants at conception, thereby theoretically mitigating the effects of confounders. Additionally, the prioritization of single-nucleotide polymorphisms (SNPs) as instrumental variables (IVs) over endpoint variations to effectively tackle the challenge of reverse causation (Gage et al., 2016). The relationship between body size and SLE remains uncertain, and no previous study has used an MR approach to investigate a corresponding causal effect.

Therefore, in this study, we aimed to investigate whether birth body size (birth weight and height) or adult body size (adult height and body mass index (BMI)) is causally associated with SLE occurrence.

## 2 Materials and methods

### 2.1 Study design

**FIGURE 1:**
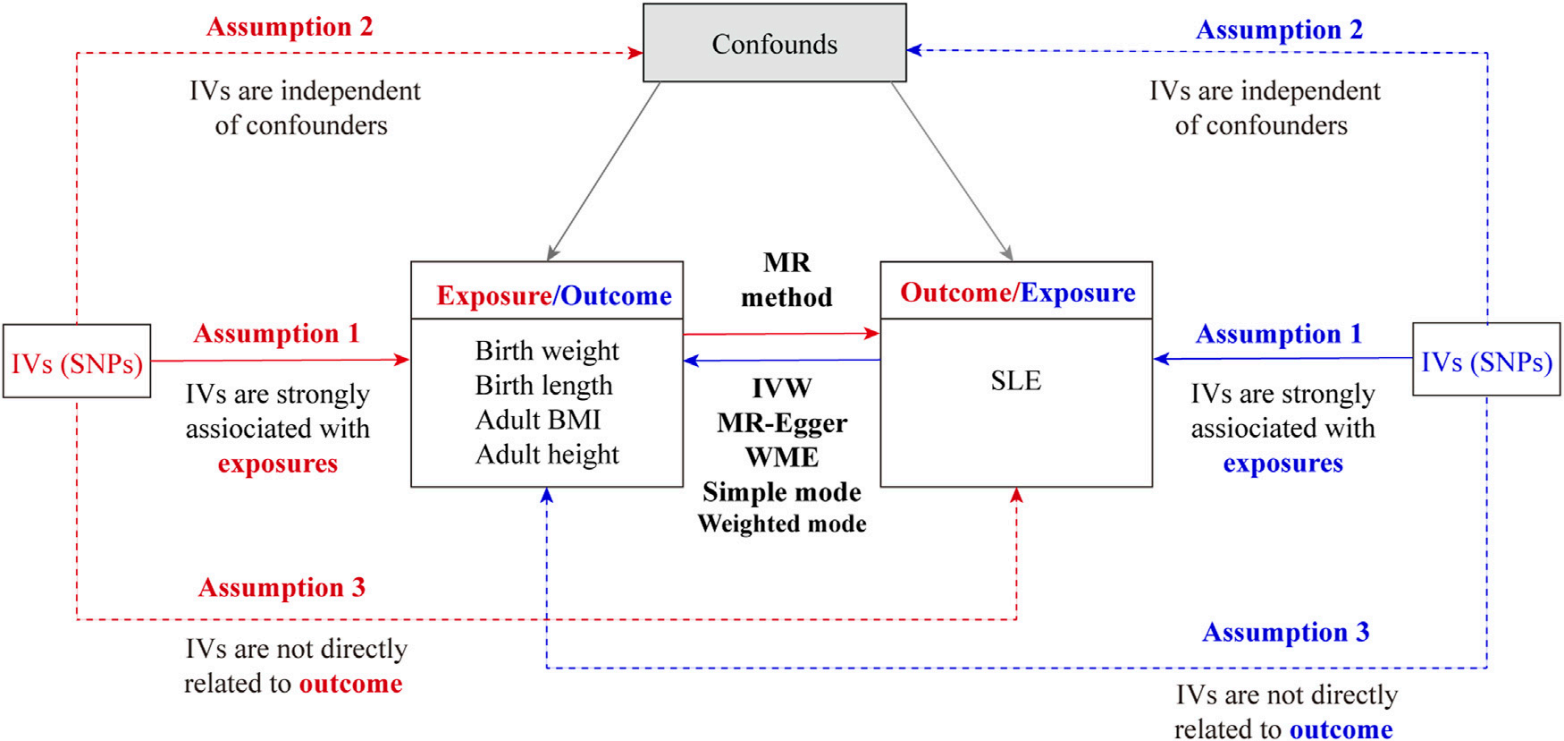
Overall flow chart of the bidirectional Mendelian randomization study. IVs, instrumental variables; SNP, single-nucleotide polymorphism; SLE, systemic lupus erythematosus; IVW, inverse variance weighting; WME, weighted median.

The bidirectional MR study design is outlined in Figure 1. Briefly, the study first estimated the causal effects of body size (birth weight, birth length, adult height, and adult BMI) on SLE, followed by evaluating the causal effects of SLE on body size. We employed SNP as an IV when three core assumptions were satisfied (Sanderson et al., 2022): 1) SNPs exhibited a strong association with exposure variables; 2) SNPs lacked any association with other known confounders; and 3) SNPs solely influenced outcomes through the exposure pathway. Assumption 1) is necessary to avoid weak instrumental variable bias when genetic variation does not strongly predict changes in exposure. If instrumental variables are correlated with potential confounders and assumption 2) is not met, we cannot be sure whether the observed associations are due to exposure factors directly or to the effects of these confounders. Assumption 3) ensures that the conclusions drawn from the analysis are based on real causal relationships and not artifacts caused by potential non-target path effects.

### 2.2 Data sources

This study was conducted using published summary statistics from the IEU Open GWAS project website (https://gwas.mrcieu.ac=/), focusing on individual European characteristics. The GWAS data for birth weight (ieu-a-1083), birth length (ieu-a-29), adult height (ieu-a-89), adult BMI (ieu-b-40), and SLE (ebi-a-GCST003156) were obtained on 31 April 2023. As GWAS data are accessible to the public and have already received ethical approval from appropriate review boards, no additional ethical authorization was required for this study. Supplementary Table S1 provides an overview of all GWASs used for MR.

### 2.3 Selection of IVs

To ensure adherence to the first assumption of MR, we specifically chose SNPs linked to birth weight, adult height, and adult BMI that demonstrated genome-wide significance, defined by a *p*-value of <5 × 10^−8^. We moderated the stringency of the threshold for selecting IVs for birth height, as no SNPs satisfied the criteria to be deemed IVs at a *p*-value of <5 × 10^−8^. Therefore, we relaxed the threshold to a *p*-value of <1 × 10^−5^. The independence of the selected SNPs was assessed by evaluating linkage disequilibrium (*r* ^2^ < 0.001; clumping window, 10,000 kb). The SNPs associated with confounders and outcomes were excluded using PhenoScanner (Assumptions 2 and 3). SNPs with F-statistics greater than 10 were chosen to minimize potential bias, and the F-statistic was calculated as F = beta^2^/se^2^. For the detection and exclusion of outliers among the instrumental variables, we performed the MR PRESSO outlier test, setting the significance level at *p* < 0.05 (Verbanck et al., 2018).

### 2.4 Statistical analysis for MR

We used the following methods to analyze the bidirectional MR between body size and SLE.

a) The primary analysis method used was inverse variance weighting (IVW) (Burgess et al., 2015), which is currently the most popular method. IVW employs the inverse of the variance (R^2^) of each locus as a weight in estimating the causal effect based on multiple SNPs as instrumental variables. It weighs the causal effect estimates for each locus and sums them to obtain a final estimate.
b) The MR-Egger (Bowden et al., 2015) method was used to detect and correct bias due to IV pleiotropy by introducing a regression intercept to estimate the causal relationship between exposure and outcome.
c) The weighted median method (Bowden et al., 2016) was selected to compute the final causal effect estimate, which involves determining the effect estimate at the 50% position after arranging all SNP effect estimates from smallest to largest.
d) A simple model (Zhu et al., 2016) was chosen to group SNPs with similar causal effect estimates according to the group with the highest number of SNPs.
e) A weighted model (Hartwig et al., 2017) was applied, which assign weights to the number of SNPs within each group based on the inverse variance of each SNP on the causal effect. This method estimates the causal effect based on the group with the highest weighted number.

### 2.5 Sensitivity analysis

To ensure the robustness of the MR analysis results, we performed a sensitivity analysis. SNP heterogeneity was determined using the Cochran’s Q test (Burgess et al., 2013); and if significant heterogeneity was noted (*p* < 0.05), random-effects IVW was used. This study identified pleiotropic effects of IVs using the MR-Egger regression and MR-PRESSO global tests (Verbanck et al., 2018). The intercept term of the MR-Egger test was statistically significant (*p* < 0.05), and the *p*-value of the MR-PRESSO global test was <0.05, indicating the presence of horizontal pleiotropy. Furthermore, a leave-one-out analysis was conducted by systematically removing each independent variable to evaluate the effects of individual SNPs on the outcomes. Funnel plots were used to visualize heterogeneity through symmetry. Scatter plots were used to depict causal effect estimates for individual variants, illustrating the associations between SNPs and outcomes in relation to SNP exposure. All the above methods were implemented using the Two-Sample MR package in the R 4.2.2 software, with a test level α = 0.05.

### 2.6 Robustness validation and secondary MR analysis

To mitigate the impact of horizontal pleiotropy, we employed the approach proposed by Long et al. to detect horizontal pleiotropy (Long et al., 2023). We sorted the SNPs in ascending order based on their MR-PRESSO outlier test *p*-values and removed them sequentially. The MR-PRESSO global test was performed on the remaining SNPs each time they were removed from the list. This recursive process was repeated until the *p*-value for the global test was no longer significant (*p* > 0.05). The remaining list of SNPs was used for a second MR analysis to validate the robustness of the results after removing pleiotropic SNPs.

## 3 Results

### 3.1 IVs for MR

Forward direction analysis was conducted to investigate the potential causal relationship between the exposure variables (birth weight, birth length, adult height, and adult BMI) and the outcome variable (SLE). After addressing the chain imbalance and confounding factors, the outlier rs11720108 was removed using the MR-PRESSO method. A total of 37 SNPs related to birth weight, 17 related to birth length, 287 related to adult height, and 395 related to adult BMI were extracted from the GWAS to serve as IVs (Supplementary Tables S2–S5). The F-statistics for individual SNPs ranged from 19.65 to 822.78, indicating a reduced risk of bias stemming from weak instrumental variables (Supplementary Table S6). We further performed a reverse MR analysis to evaluate whether SLE had a causal effect on body size. We screened for SNPs that met the genome-wide significance threshold (*p* < 5 × 10^−8^). The MR-PRESSO test identified and excluded five outliers. Detailed information, including the number of SNPs and distribution of F-statistics for individual SNPs, is provided in Supplementary Tables S7–S11.

### 3.2 MR results

**FIGURE 2:**
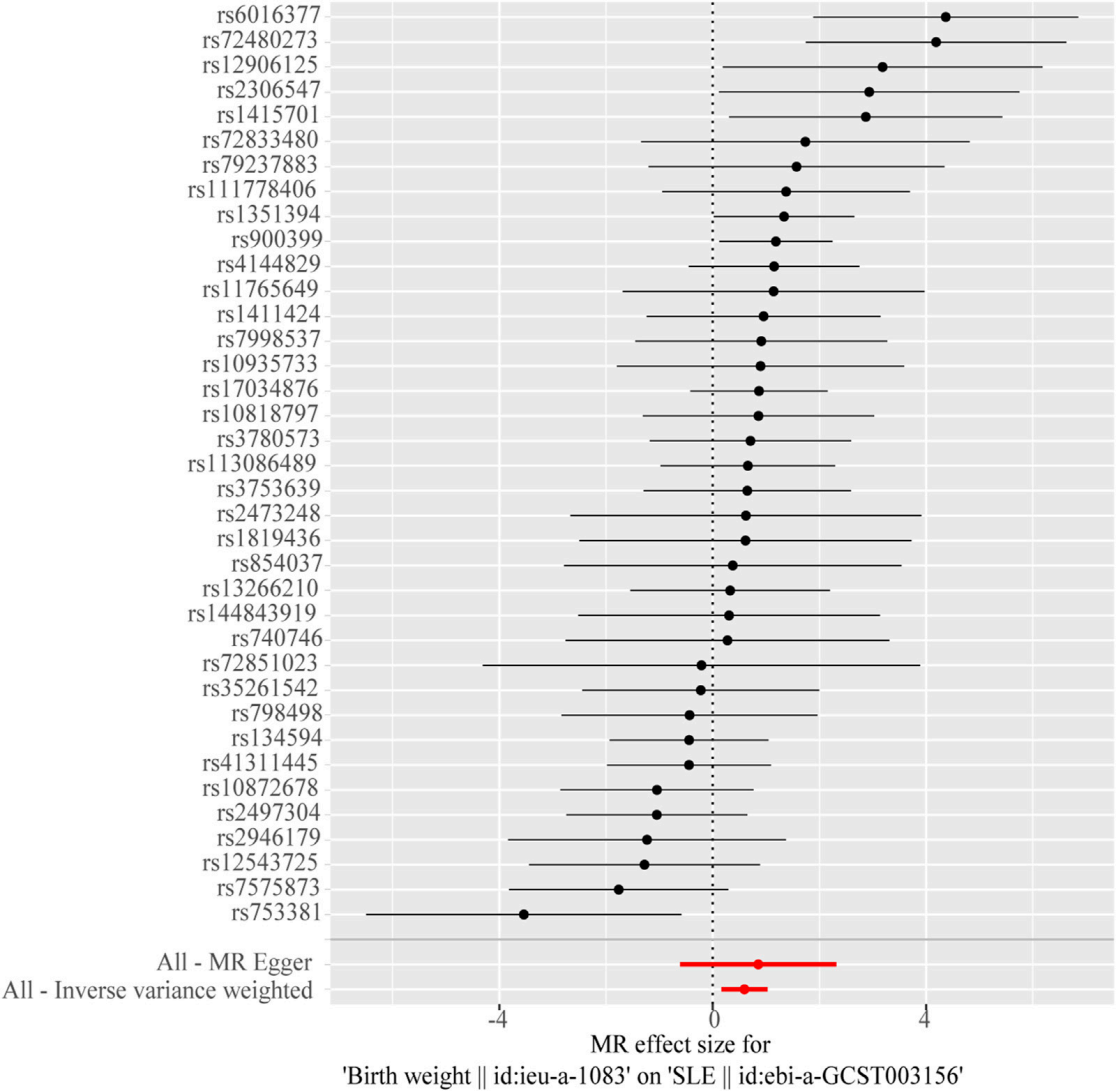
Forest plot of the causal effects of single-nucleotide polymorphisms (SNPs) associated with birth weight on systemic lupus erythematosus.

**FIGURE 3:**
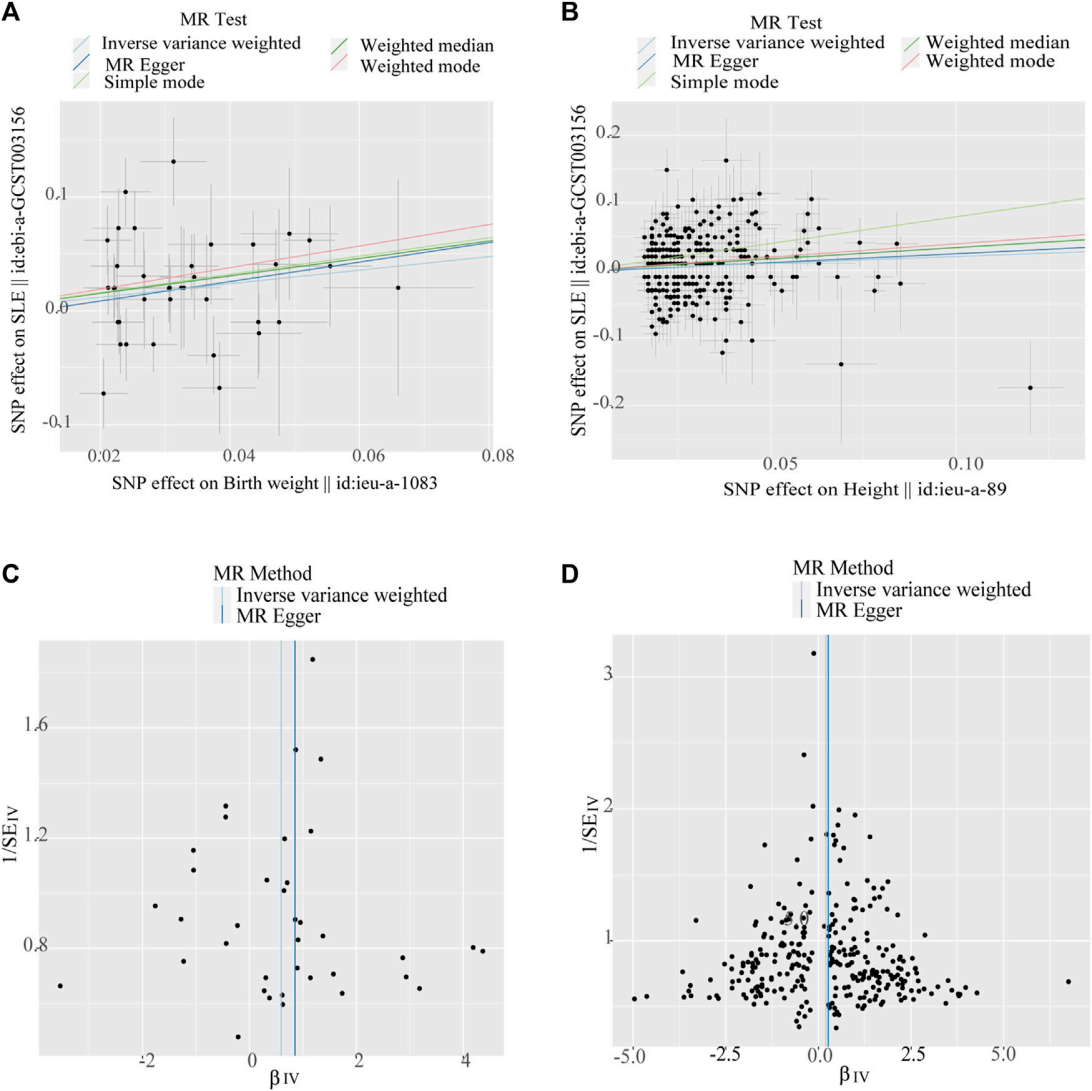
Scatter plots and funnel plots of single-nucleotide polymorphisms (SNPs). **(A)** Scatter plot of genetic associations with birth weight against the genetic associations with systemic lupus erythematosus (SLE). **(B)** Scatter plot of genetic associations with height against the genetic associations with SLE. **(C)** Funnel plot to assess heterogeneity in birth weight associated with single-nucleotide polymorphisms SNPs. **(D)** Funnel plot to assess heterogeneity in adult height-associated SNPs.

The IVW model results showed a positive causal connection between birth weight and SLE (OR, 1.811; 95% CI, 1.174–2.793; *p* < 0.05; Table 1 and Figure 2). A positive causal relationship between adult height and SLE was also observed (OR, 1.225; 95% CI, 1.046–1.434; *p* < 0.05; Table 1; Supplementary Figure S1). In addition, the MR results of MR-Egger, weighted median, simple mode, and weighted mode remained parallel to the IVW results (Table 1; Figures 3A, B). However, there was no evidence to support a causal relationship between birth length, adult BMI, and SLE (Table 1).

**TABLE 1:** Mendelian randomization estimates from each method of assessing the causal effect of body size on the risk of systemic lupus erythematosus (forward direction).

Exposure	*n* SNPs	Method	*β*	*SE*	OR (95% CI)	*p*-value
Birth weight	37	IVW	0.594	0.221	1.811 (1.174–2.793)	0.007
		Weighted median	0.766	0.267	2.150 (1.274–3.629)	0.004
		MR Egger	0.853	0.747	2.348 (0.543–10.144)	0.261
		Simple mode	0.800	0.503	2.227 (0.831–5.964)	0.120
		Weighted mode	0.947	0.417	2.578 (1.138–5.837)	0.029
Birth length	17	IVW	0.074	0.169	1.077 (0.772–1.502)	0.663
		Weighted median	0.109	0.205	1.115 (0.746–1.666)	0.596
		MR Egger	−0.322	0.787	0.725 (0.155–3.386)	0.688
		Simple mode	−0.040	0.352	0.961 (0.482–1.914)	0.911
		Weighted mode	−0.072	0.302	0.931 (0.514–1.684)	0.815
Height	287	IVW	0.203	0.081	1.225 (1.046–1.434)	0.012
		Weighted median	0.334	0.109	1.397 (1.129–1.728)	0.002
		MR Egger	0.265	0.214	1.303 (0.856–1.983)	0.218
		Simple mode	0.791	0.340	2.206 (1.132–4.298)	0.021
		Weighted mode	0.390	0.220	1.478 (0.959–2.276)	0.078
BMI	395	IVW	0.045	0.124	1.046 (0.821–1.333)	0.718
		Weighted median	−0.139	0.181	0.870 (0.610–1.242)	0.444
		MR Egger	0.504	0.329	1.655 (0.869–3.155)	0.126
		Simple mode	1.508	0.493	4.520 (1.718–11.888)	0.002
		Weighted mode	−0.140	0.247	0.870 (0.536–1.410)	0.571

MR, mendelian randomization; SNP, single-nucleotide polymorphism; *β*, beta coefficient; *SE*, standard error; OR, odds ratio; CI, confidence interval; IVW, inverse variance weighting.

**FIGURE 4:**
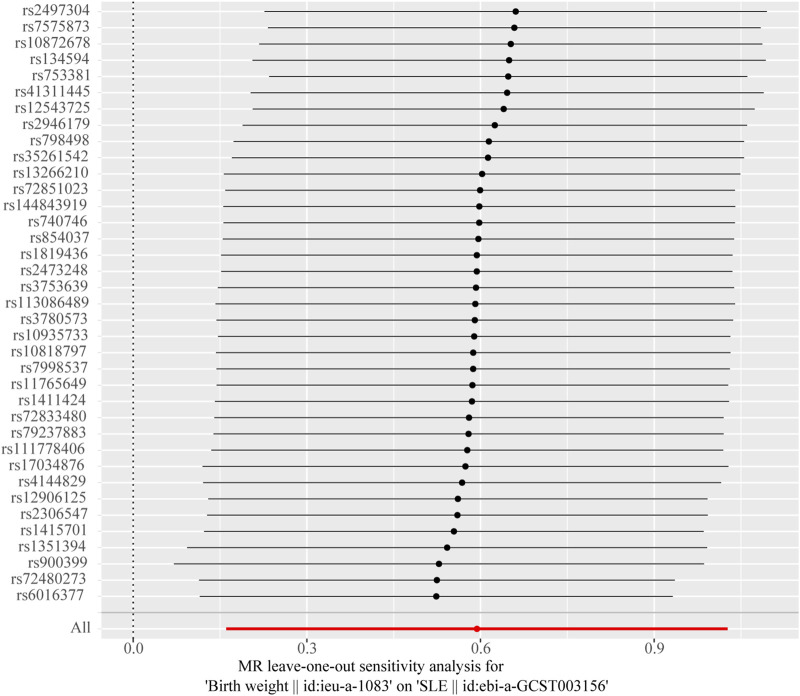
Leave-one-out sensitivity analysis to investigate the possibility that the causal association was driven by a unique single-nucleotide polymorphism associated with birth weight in systemic lupus erythematosus.

The Cochran’s Q test from the MR-Egger regression and IVW models indicated significant heterogeneity between birth weight, adult height, and the risk of SLE (Table 2). Therefore, the random-effects IVW method was used to mitigate the influence of heterogeneity. However, the scatter plots for birth weight (Figure 3A) and adult height (Figure 3B) showed a symmetric distribution and close clustering of all the included SNPs. Funnel plots for birth weight (Figure 3C) and adult height (Figure 3D) were symmetrically distributed and formed an inverted funnel shape. These observations suggest that a potential bias is less likely to affect causal associations. The difference between the MR-Egger regression intercept term and zero was not statistically significant (*p* > 0.05), indicating no evidence of pleiotropy among the SNPs associated with birth weight and adult height (Table 2). The MR-PRESSO global tests showed statistical significance (*p* < 0.05; Table 2). Leave-one-out analyses uncovered association between genetically predicted birth weight (Figure 4) and adult height (Supplementary Figure S2), with no discernable impact on the risk of SLE attributable to any individual SNP.

**TABLE 2:** Results of heterogeneity and pleiotropy analyses of body size in systemic lupus erythematosus (forward direction).

Exposure	*n* SNPs	Method	Heterogeneity test		Pleiotropy test			
					MR Egger			MR-PRESSO *p-*value
			I^2^	Cochran’s *p*-value	Intercept	*SE*	*p-*value	
Birth weight	37	MR Egger	41	0.006	−0.009	0.023	0.718	0.01
		IVW	40	0.007				
Birth length	17	MR Egger	35	0.081	0.022	0.042	0.613	0.123
		IVW	32	0.099				
Height	287	MR Egger	40	<0.001	−0.002	0.007	0.756	<0.001
		IVW	40	<0.001				
BMI	395	MR Egger	36	<0.001	−0.008	0.005	0.133	<0.001
		IVW	37	<0.001				

SNP, single-nucleotide polymorphism; SE, standard error; IVW, inverse variance weighting; BMI, body mass index; MR, mendelian randomization; MR-PRESSO, Mendelian Randomization Pleiotropy RESidual Sum and Outlier.

### 3.3 Results of the reverse MR analysis

**FIGURE 5:**
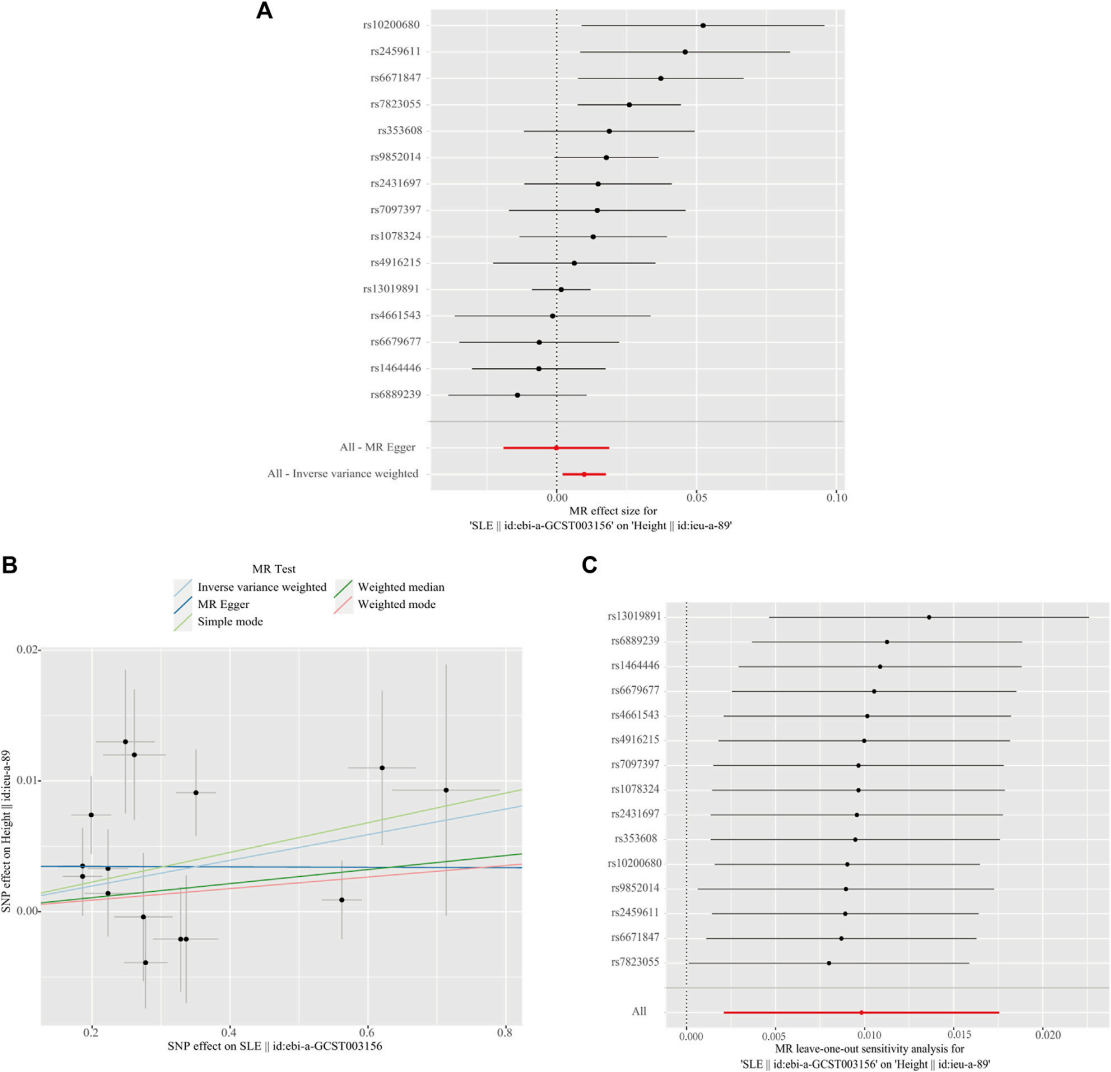
Reverse direction Mendelian randomization analysis. **(A)** Forest plot of the causal effects of single-nucleotide polymorphisms (SNPs) associated with systemic lupus erythematosus (SLE) on height. **(B)** Scatter plot of genetic associations with SLE against the genetic associations with height. **(C)** Leave-one-out sensitivity analysis was conducted to investigate the possibility that the causal association was driven by a unique SNP associated with SLE on height.

We performed MR analysis in the reverse direction to investigate the effect of SLE on birth weight, birth length, adult BMI, and adult height. We identified SLE as a weak causal factor for increased height (IVW, OR, 1.010; 95% CI, 1.002–1.018; *p* < 0.05; Table 3 and Figure 5A). However, no causal relationships were found between SLE and birth weight, birth length, or adult BMI (Table 3). The Cochran’s Q test showed that the reverse MR analysis was influenced by heterogeneity (*p* < 0.05) (Table 4). However, the height scatter plot (Figure 5B) indicated that the direction of the impact was generally consistent. Furthermore, the MR-Egger intercept test suggested that the reverse MR analysis was not influenced by water product pleiotropy (*p* > 0.05; Table 4). The MR-PRESSO global tests showed statistical significance (*p* < 0.05; Table 4). Finally, leave-one-out sensitivity analysis confirmed the robustness of the reverse MR results (Figure 5C).

**TABLE 3:** Mendelian randomization analysis of systemic lupus erythematosus on birth weight, birth length, adult height, and body mass index (reverse direction).

Outcome	*n* SNPs	Method	*β*	*SE*	OR (95% CI)	*p*-value
Birth weight	39	IVW	−0.0004	0.002	0.999 (0.995–1.004)	0.861
		Weighted median	0.0002	0.004	1.000 (0.993–1.007)	0.944
		MR Egger	0.004	0.005	1.004 (0.994–1.014)	0.467
		Simple mode	0.001	0.007	1.001 (0.987–1.015)	0.903
		Weighted mode	0.001	0.005	1.001 (0.991–1.010)	0.861
Birth length	16	IVW	−0.001	0.008	0.999 (0.982–1.016)	0.891
		Weighted median	−0.003	0.012	0.996 (0.974–1.019)	0.757
		MR Egger	0.008	0.018	1.008 (0.974–1.044)	0.650
		Simple mode	−0.003	0.017	0.997 (0.964–1.031)	0.878
		Weighted mode	−0.003	0.014	0.997 (0.969–1.026)	0.854
Height	15	IVW	0.010	0.004	1.010 (1.002–1.018)	0.013
		Weighted median	0.005	0.004	1.005 (0.997–1.014)	0.230
		MR Egger	−0.0001	0.009	0.999 (0.981–1.019)	0.989
		Simple mode	0.011	0.009	1.011 (0.993–1.030)	0.248
		Weighted mode	0.004	0.005	1.004 (0.995–1.014)	0.386
BMI	11	IVW	−0.002	0.002	0.998 (0.994–1.003)	0.459
		Weighted median	−0.0002	0.003	0.999 (0.994–1.005)	0.936
		MR Egger	−0.005	0.006	0.995 (0.983–1.006)	0.398
		Simple mode	−0.001	0.005	0.999 (0.989–1.009)	0.839
		Weighted mode	−0.0003	0.005	0.999 (0.991–1.009)	0.950

MR, mendelian randomization; SNP, single-nucleotide polymorphism; *β*, beta coefficient; *SE*, standard error; OR, odds ratio; CI, confidence interval; IVW, inverse variance weighting; BMI, body mass index.

**TABLE 4:** Heterogeneity and pleiotropy analyses of systemic lupus erythematosus on body size (reverse direction).

Outcome	*n* SNPs	Method	Heterogeneity test		Pleiotropy test			
					MR Egger			MR-PRESSO *p*-value
			I^2^	Cochran’s *p*-value	Intercept	*SE*	*p*-value	
Birth weight	39	MR Egger	16	0.197	−0.002	0.002	0.359	0.194
		IVW	16	0.198				
Birth length	16	MR Egger	2	0.427	−0.004	0.007	0.557	0.490
		IVW	0	0.475				
Height	15	MR Egger	41	0.055	0.003	0.003	0.279	0.046
		IVW	42	0.044				
BMI	11	MR Egger	5	0.397	0.001	0.002	0.524	0.461
		IVW	0	0.449				

SNP, single-nucleotide polymorphism; SE, standard error; IVW, inverse variance weighting; BMI, body mass index; MR, Mendelian randomization; MR-PRESSO, Mendelian Randomization Pleiotropy RESidual Sum and Outlier.

### 3.4 Robustness validation and secondary MR analysis

Pleiotropy analyses were performed using MR-Egger and MR-PRESSO global tests, which yielded different results. MR-Egger suggested no horizontal pleiotropy, whereas MR-PRESSO global did. The list of SNPs remaining after the removal of the pleiotropic SNPs was used for a second MR analysis to confirm the robustness of the results. Even when heterogeneity and pleiotropy analyses showed no heterogeneity or pleiotropy among the instrumental variables, we still found that birth weight (OR, 1.571; 95% CI, 1.074–2.298; *p* = 0.020) and height (OR, 1.448; 95% CI, 1.036–2.024; *p* = 0.030) were risk factors for SLE, and SLE was a positive causal factor for height (OR, 1.013; 95% CI, 1.005–1.023; *p* = 0.003). The conclusions of the secondary MR analysis were consistent with previous findings (Table 5).

**TABLE 5:** Secondary Mendelian randomization analysis (bidirectional).

Exposure	Outcome	*n* SNPs	Method	OR (95% CI)	*p*-value	Heterogeneity test		Pleiotropy test	
						I^2^	Cochran’s *p*-value	MR Egger	MR-PRESSO
Birth weight	SLE	35	IVW	1.571 (1.074–2.298)	0.020	19	0.161	0.354	0.168
			MR Egger	2.790 (0.734–9.809)	0.119	19	0.160		
Birth length	SLE	17	IVW	1.077 (0.772–1.502)	0.663	35	0.099	0.613	0.123
			MR Egger	0.725 (0.155–3.386)	0.688	32	0.081		
Height	SLE	53	IVW	1.448 (1.036–2.024)	0.030	23	0.061	0.213	0.058
			MR Egger	2.526 (0.999–6.380)	0.055	24	0.070		
BMI	SLE	208	IVW	0.856 (0.632–1.158)	0.313	14	0.052	0.323	0.050
			MR Egger	1.288 (0.543–3.056)	0.566	14	0.052		
SLE	Birth weight	39	IVW	0.999 (0.995–1.004)	0.861	16	0.197	0.359	0.194
			MR Egger	1.004 (0.994–1.014)	0.467	16	0.199		
SLE	Birth length	16	IVW	0.999 (0.982–1.016)	0.891	0	0.475	0.557	0.490
			MR Egger	1.008 (0.974–1.044)	0.650	2	0.427		
SLE	Height	14	IVW	1.013 (1.005–1.023)	0.003	37	0.079	0.719	0.104
			MR Egger	1.009 (0.982–1.036)	0.524	41	0.058		
SLE	BMI	11	IVW	0.998 (0.994–1.003)	0.459	0	0.449	0.524	0.502
			MR Egger	0.995 (0.983–1.006)	0.398	5	0.397		

SNP, single-nucleotide polymorphism; OR, odds ratio; CI, confidence interval; IVW, inverse variance weighting; BMI, body mass index; MR, Mendelian randomization; MR-PRESSO, Mendelian Randomization Pleiotropy RESidual Sum and Outlier.

## 4 Discussion

Using various MR methods, this bidirectional MR investigation identified a positive causal relationship between birth weight, adult height, and SLE occurrence. Reverse MR analysis revealed that SLE had a weak causal effect on adult height. Furthermore, sensitivity analysis revealed the robustness of the causal relationships. To the best of our knowledge, this is the first study to investigate the relationship between birth body size and SLE using a comprehensive GWAS summary dataset for MR analysis, thereby improving and refining the findings of previous studies.

Previous studies have reported different associations between birth weight and the incidence of SLE. In an earlier case–control study, American researchers found no significant differences in birth weight or length between patients with SLE and healthy controls (Coleman et al., 2005). Similarly, a Swedish nested case–control study did not identify high birth weight as a risk factor for SLE (Arkema and Simard, 2015). Transitioning to retrospective cohort designs, a study revealed an association between SLE and low birth weight (<2,500 g vs. <3,000–3,500 g; OR, 2.2; 95% CI, 1.2–3.9) (Parks et al., 2016). By contrast, a prospective cohort study showed findings consistent with the results we obtained herein; their study, which spanned 26 years, found a positive association between a birth weight of ≥10 pounds and the incidence of SLE in women (rate ratio [RR], 2.7; 95% CI, 1.2–5.9) (Simard et al., 2008). These contradictory findings may be related to variations in study design, study population, and case identification procedures. Although observational clinical studies cannot establish direct causal relations, stronger evidence combined with a more comprehensive sample size can help us understand the true causative connection.

In a large Danish cohort, birth weight was not associated with the risk of SLE; however, childhood BMI and height were linearly associated with the risk of developing adult SLE (Thomas et al., 2020). This increased risk persists even when a healthy body size is achieved in adulthood (Zhao et al., 2022). A polygenic risk score for SLE, consisting of 36 SNPs, has recently been associated with adult sitting and standing heights (Richardson et al., 2019). The mechanisms underlying these findings may be attributed to the developmental plasticity of the physical condition at birth and the early immune system. The cumulative effects of growth and development may gradually manifest in the future, ultimately leading to the development of SLE (Edwards and Cooper, 2006). These findings are consistent with the results of the forward analysis. Our study provides additional evidence through MR methods that height increases susceptibility to SLE. Using SNP as instrumental variables in MR research can mitigate the potential interference from acquired exposure variables on the causal relationship between height and SLE onset.

Our reverse analysis revealed a potential positive correlation between SLE and increased height, corroborated by evidence from previous observational studies. A multicenter case-control spanning 2 years study demonstrated that adult patients with SLE were taller than age-matched healthy controls (Zhu et al., 2015). Notably, the age at SLE onset appears to be closely related to the final height. Patients who developed SLE during childhood are shorter than those who developed it during adulthood (Heshin-Bekenstein et al., 2018). Female patients who developed SLE before menarche are shorter than those who developed SLE after menarche (Sontichai et al., 2022). Research focusing on children with SLE has also identified sex and hormone use as factors that lead to reduced final height in patients with SLE (Jongvilaikasem and Rianthavorn, 2021; Ponin et al., 2022). A study on bone remodeling in children and youths with SLE showed that the dose of glucocorticoids was a negative predictor of bone formation, whereas SLE disease activity was unrelated to bone formation (Baker-LePain et al., 2011). Genes remain constant throughout life. Therefore, the results of MR analyses are often interpreted as the average lifelong effects of the genetic predisposition to exposure (Power et al., 2023). In the present study, we found that SLE was a weak positive factor for height in terms of average lifelong effects. However, considering the effect of age at SLE onset on height and the small OR (OR = 1.010) from the MR analysis, we must be cautious when interpreting the positive correlation between SLE and height in patients of different ages at disease onset.

Employing SNPs as instrumental variables leverage their formation during the embryonic gamete stage, ensuring random assignment and combination. Using MR to explore the causal relationship between early-life and adult body size concerning SLE occurrence mitigates the issue of reverse causality. Given the multifactorial nature of SLE onset, potential confounders influencing the outcomes were meticulously excluded to ensure validity of the results. In addition, we assessed the combined effects of multiple SNPs strongly associated with birth weight and adult height, thereby providing a strong genetic instrument. Third, we obtained a large sample of GWAS data related to height, birth weight, birth length, and SLE; therefore, the statistical power was high in most analyses. At the same time, the research sample was limited to the European population, and population stratification bias was minimized.

Our analysis has some limitations. Because the threshold for birth length-related SNPs as an instrumental variable was set at *p*-value < 5 × 10^−8^ and no SNPs met the criteria, we relaxed the threshold criterion to *p*-value < 1 × 10^−5^. Although we did not identify a causal relationship between birth length and SLE, a relaxation in the threshold may have influenced the conclusion regarding this relationship, necessitating further investigation. Furthermore, due to the use of summary statistics, we were unable to assess the independence of exposure-outcome connection confounders from the IVs. Subgroup testing is only possible when individual-level data is available. For instance, because there are differences between the sexes in both body size and SLE development, it may be necessary to conduct MR analysis separately in females and males to reveal sex-specific estimates. Another limitation of our analysis is that our study dataset was based on European populations; unfortunately, an East Asian dataset of exposure variables was missing. For example, the birth length dataset of East Asian populations was not queried in the IEU Open GWAS, GWAS Catalog, or FinnGen. Therefore, an East Asian population-based analysis was not possible. Therefore, the applicability of our findings to people from other regions requires further exploration.

This study provides evidence of a causal link among birth weight, adult height, and SLE. Our findings suggest that high birth weight and adult height may contribute to an increased risk of developing SLE. In the reverse analysis, SLE as an exposure factor was a weak causal factor for adult height. However, no evidence has been found to establish a causal link between birth length, adult BMI, and SLE.

## Data Availability

The original contributions presented in the study are included in the article/Supplementary Material, further inquiries can be directed to the corresponding author.
