# Supplementary figures and images for: Causal associations of birth body size and adult body size with systemic lupus erythematosus: a bidirectional mendelian randomization study
Source: Front Genet. 2024 May 6;15:1368497. doi: 10.3389/fgene.2024.1368497 (PMC11102996; doi:10.3389/fgene.2024.1368497)

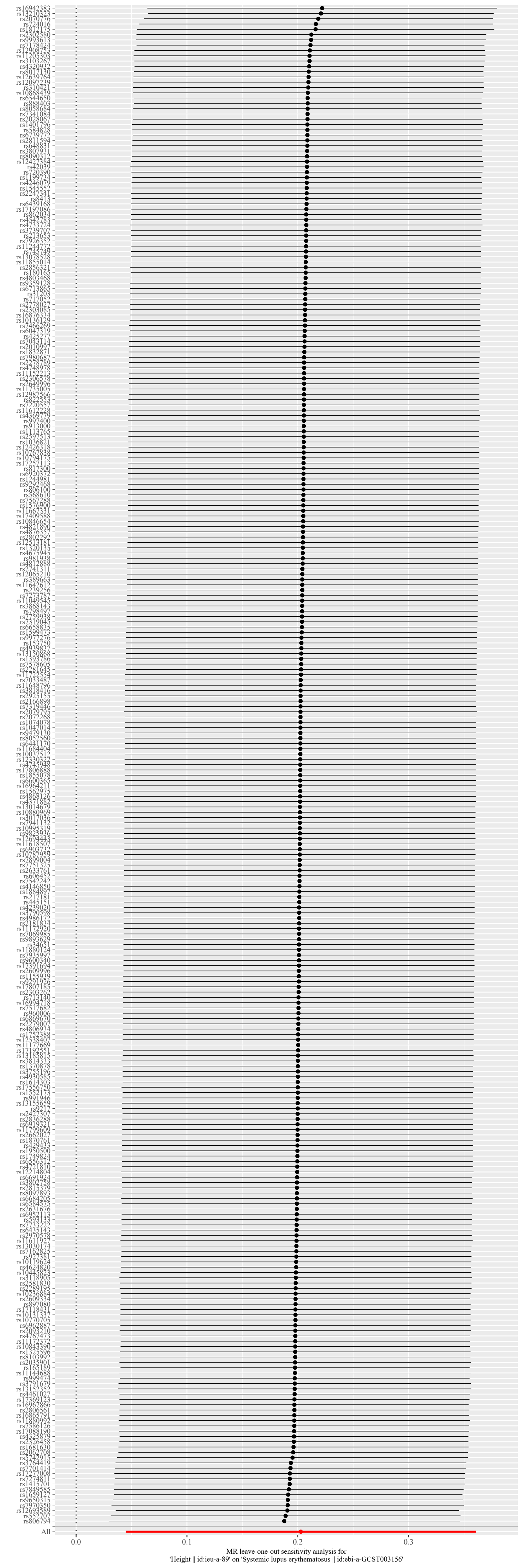

Supplement: Supplementary file 1 [file DataSheet2.PDF]

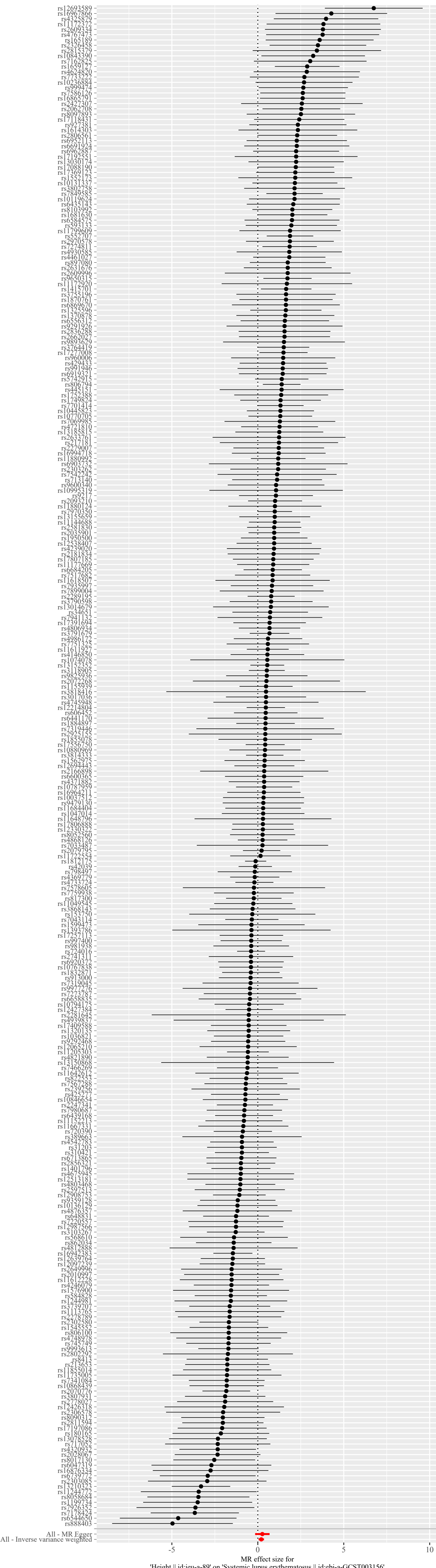

Supplement: Supplementary file 3 [file DataSheet1.PDF]
